# Supplementary material for: HIVprotI: an integrated web based platform for prediction and design of HIV proteins inhibitors
Source: J Cheminform. 2018 Mar 9;10:12. doi: 10.1186/s13321-018-0266-y (PMC5845081; doi:10.1186/s13321-018-0266-y)
Supplement: Supplementary file 2 — Additional file 2. Source code of HIVProtI web server. [file 13321_2018_266_MOESM2_ESM.zip › HIVprotI_Source-code/hivprotI/weka-3-6-6/doc/weka/filters/unsupervised/instance/RemoveWithValues.html]

RemoveWithValues


---


|  |  |  |
| --- | --- | --- |
| **Overview** \| **Package** \| **Class** \| **Tree** \| **Deprecated** \| **Index** \| **Help** \| **Weka's home** | |  |
| **PREV CLASS**   **NEXT CLASS** | **FRAMES**    **NO FRAMES**     **All Classes** |  |
| SUMMARY: NESTED \| FIELD \| CONSTR \| METHOD | DETAIL: FIELD \| CONSTR \| METHOD |  |


---


## weka.filters.unsupervised.instance Class RemoveWithValues

```
java.lang.Object
  weka.filters.Filter
      weka.filters.unsupervised.instance.RemoveWithValues
```

**All Implemented Interfaces:**: java.io.Serializable, CapabilitiesHandler, OptionHandler, RevisionHandler, StreamableFilter, UnsupervisedFilter

---

``` public class RemoveWithValues extends Filter implements UnsupervisedFilter, StreamableFilter, OptionHandler ```

Filters instances according to the value of an attribute.


Valid options are:

```
 -C <num>
  Choose attribute to be used for selection.
```

```
 -S <num>
  Numeric value to be used for selection on numeric
  attribute.
  Instances with values smaller than given value will
  be selected. (default 0)
```

```
 -L <index1,index2-index4,...>
  Range of label indices to be used for selection on
  nominal attribute.
  First and last are valid indexes. (default all values)
```

```
 -M
  Missing values count as a match. This setting is
  independent of the -V option.
  (default missing values don't match)
```

```
 -V
  Invert matching sense.
```

```
 -H
  When selecting on nominal attributes, removes header
  references to excluded values.
```

```
 -F
  Do not apply the filter to instances that arrive after the first
  (training) batch. The default is to apply the filter (i.e.
  the filter may not return an instance if it matches the remove criteria)
```

**Version:**
:   $Revision: 7602 $

**Author:**
:   Eibe Frank (eibe@cs.waikato.ac.nz)

**See Also:**: Serialized Form

---

**Constructor Summary**

`RemoveWithValues()`
  
          Default constructor


| **Method Summary** | |
| --- | --- |
| `java.lang.String` | `attributeIndexTipText()`             Returns the tip text for this property |
| `java.lang.String` | `dontFilterAfterFirstBatchTipText()`             Returns the tip text for this property. |
| `java.lang.String` | `getAttributeIndex()`             Get the index of the attribute used. |
| `Capabilities` | `getCapabilities()`             Returns the Capabilities of this filter. |
| `boolean` | `getDontFilterAfterFirstBatch()`             Get whether to apply the filter to instances that arrive once the first (training) batch has been seen. |
| `boolean` | `getInvertSelection()`             Get whether the supplied columns are to be removed or kept |
| `boolean` | `getMatchMissingValues()`             Gets whether missing values are counted as a match. |
| `boolean` | `getModifyHeader()`             Gets whether the header will be modified when selecting on nominal attributes. |
| `java.lang.String` | `getNominalIndices()`             Get the set of nominal value indices that will be used for selection |
| `java.lang.String[]` | `getOptions()`             Gets the current settings of the filter. |
| `java.lang.String` | `getRevision()`             Returns the revision string. |
| `double` | `getSplitPoint()`             Get the split point used for numeric selection |
| `java.lang.String` | `globalInfo()`             Returns a string describing this classifier |
| `boolean` | `input(Instance instance)`             Input an instance for filtering. |
| `java.lang.String` | `invertSelectionTipText()`             Returns the tip text for this property |
| `boolean` | `isNominal()`             Returns true if selection attribute is nominal. |
| `boolean` | `isNumeric()`             Returns true if selection attribute is numeric. |
| `java.util.Enumeration` | `listOptions()`             Returns an enumeration describing the available options. |
| `static void` | `main(java.lang.String[] argv)`             Main method for testing this class. |
| `java.lang.String` | `matchMissingValuesTipText()`             Returns the tip text for this property |
| `java.lang.String` | `modifyHeaderTipText()`             Returns the tip text for this property |
| `java.lang.String` | `nominalIndicesTipText()`             Returns the tip text for this property |
| `void` | `setAttributeIndex(java.lang.String attIndex)`             Sets index of the attribute used. |
| `void` | `setDontFilterAfterFirstBatch(boolean b)`             Set whether to apply the filter to instances that arrive once the first (training) batch has been seen. |
| `boolean` | `setInputFormat(Instances instanceInfo)`             Sets the format of the input instances. |
| `void` | `setInvertSelection(boolean invert)`             Set whether selected values should be removed or kept. |
| `void` | `setMatchMissingValues(boolean newMatchMissingValues)`             Sets whether missing values are counted as a match. |
| `void` | `setModifyHeader(boolean newModifyHeader)`             Sets whether the header will be modified when selecting on nominal attributes. |
| `void` | `setNominalIndices(java.lang.String rangeList)`             Set which nominal labels are to be included in the selection. |
| `void` | `setNominalIndicesArr(int[] values)`             Set which values of a nominal attribute are to be used for selection. |
| `void` | `setOptions(java.lang.String[] options)`             Parses a given list of options. |
| `void` | `setSplitPoint(double value)`             Split point to be used for selection on numeric attribute. |
| `java.lang.String` | `splitPointTipText()`             Returns the tip text for this property |

**Methods inherited from class weka.filters.Filter**

`batchFilterFile, batchFinished, filterFile, getCapabilities, getOutputFormat, isFirstBatchDone, isNewBatch, isOutputFormatDefined, makeCopies, makeCopy, numPendingOutput, output, outputPeek, toString, useFilter, wekaStaticWrapper`

**Methods inherited from class java.lang.Object**

`equals, getClass, hashCode, notify, notifyAll, wait, wait, wait`

**Constructor Detail**

### RemoveWithValues

```
public RemoveWithValues()
```

:   Default constructor


**Method Detail**

### globalInfo

```
public java.lang.String globalInfo()
```

:   Returns a string describing this classifier

    :   **Returns:**: a description of the classifier suitable for displaying in the explorer/experimenter gui

---


### listOptions

```
public java.util.Enumeration listOptions()
```

:   Returns an enumeration describing the available options.

    :   **Specified by:**: `listOptions` in interface `OptionHandler`
    :   **Returns:**: an enumeration of all the available options.

---


### setOptions

```
public void setOptions(java.lang.String[] options)
                throws java.lang.Exception
```

:   Parses a given list of options.


    Valid options are:

    ```
     -C <num>
      Choose attribute to be used for selection.
    ```

    ```
     -S <num>
      Numeric value to be used for selection on numeric
      attribute.
      Instances with values smaller than given value will
      be selected. (default 0)
    ```

    ```
     -L <index1,index2-index4,...>
      Range of label indices to be used for selection on
      nominal attribute.
      First and last are valid indexes. (default all values)
    ```

    ```
     -M
      Missing values count as a match. This setting is
      independent of the -V option.
      (default missing values don't match)
    ```

    ```
     -V
      Invert matching sense.
    ```

    ```
     -H
      When selecting on nominal attributes, removes header
      references to excluded values.
    ```

    ```
     -F
      Do not apply the filter to instances that arrive after the first
      (training) batch. The default is to apply the filter (i.e.
      the filter may not return an instance if it matches the remove criteria)
    ```

    :   **Specified by:**: `setOptions` in interface `OptionHandler`
    :   **Parameters:**: `options` - the list of options as an array of strings **Throws:**: `java.lang.Exception` - if an option is not supported

---


### getOptions

```
public java.lang.String[] getOptions()
```

:   Gets the current settings of the filter.

    :   **Specified by:**: `getOptions` in interface `OptionHandler`
    :   **Returns:**: an array of strings suitable for passing to setOptions

---


### getCapabilities

```
public Capabilities getCapabilities()
```

:   Returns the Capabilities of this filter.

    :   **Specified by:**: `getCapabilities` in interface `CapabilitiesHandler` **Overrides:**: `getCapabilities` in class `Filter`
    :   **Returns:**: the capabilities of this object **See Also:**: `Capabilities`

---


### setInputFormat

```
public boolean setInputFormat(Instances instanceInfo)
                       throws java.lang.Exception
```

:   Sets the format of the input instances.

    :   **Overrides:**: `setInputFormat` in class `Filter`
    :   **Parameters:**: `instanceInfo` - an Instances object containing the input instance structure (any instances contained in the object are ignored - only the structure is required). **Returns:**: true because outputFormat can be collected immediately **Throws:**: `UnsupportedAttributeTypeException` - if the specified attribute is neither numeric or nominal.: `java.lang.Exception` - if the inputFormat can't be set successfully

---


### input

```
public boolean input(Instance instance)
```

:   Input an instance for filtering. Ordinarily the instance is processed
    and made available for output immediately. Some filters require all
    instances be read before producing output.

    :   **Overrides:**: `input` in class `Filter`
    :   **Parameters:**: `instance` - the input instance **Returns:**: true if the filtered instance may now be collected with output(). **Throws:**: `java.lang.IllegalStateException` - if no input format has been set.

---


### isNominal

```
public boolean isNominal()
```

:   Returns true if selection attribute is nominal.

    :   **Returns:**: true if selection attribute is nominal

---


### isNumeric

```
public boolean isNumeric()
```

:   Returns true if selection attribute is numeric.

    :   **Returns:**: true if selection attribute is numeric

---


### modifyHeaderTipText

```
public java.lang.String modifyHeaderTipText()
```

:   Returns the tip text for this property

    :   **Returns:**: tip text for this property suitable for displaying in the explorer/experimenter gui

---


### getModifyHeader

```
public boolean getModifyHeader()
```

:   Gets whether the header will be modified when selecting on nominal
    attributes.

    :   **Returns:**: true if so.

---


### setModifyHeader

```
public void setModifyHeader(boolean newModifyHeader)
```

:   Sets whether the header will be modified when selecting on nominal
    attributes.

    :   **Parameters:**: `newModifyHeader` - true if so.

---


### attributeIndexTipText

```
public java.lang.String attributeIndexTipText()
```

:   Returns the tip text for this property

    :   **Returns:**: tip text for this property suitable for displaying in the explorer/experimenter gui

---


### getAttributeIndex

```
public java.lang.String getAttributeIndex()
```

:   Get the index of the attribute used.

    :   **Returns:**: the index of the attribute

---


### setAttributeIndex

```
public void setAttributeIndex(java.lang.String attIndex)
```

:   Sets index of the attribute used.

    :   **Parameters:**: `attIndex` - the index of the attribute

---


### splitPointTipText

```
public java.lang.String splitPointTipText()
```

:   Returns the tip text for this property

    :   **Returns:**: tip text for this property suitable for displaying in the explorer/experimenter gui

---


### getSplitPoint

```
public double getSplitPoint()
```

:   Get the split point used for numeric selection

    :   **Returns:**: the numeric split point

---


### setSplitPoint

```
public void setSplitPoint(double value)
```

:   Split point to be used for selection on numeric attribute.

    :   **Parameters:**: `value` - the split point

---


### matchMissingValuesTipText

```
public java.lang.String matchMissingValuesTipText()
```

:   Returns the tip text for this property

    :   **Returns:**: tip text for this property suitable for displaying in the explorer/experimenter gui

---


### getMatchMissingValues

```
public boolean getMatchMissingValues()
```

:   Gets whether missing values are counted as a match.

    :   **Returns:**: true if missing values are counted as a match.

---


### setMatchMissingValues

```
public void setMatchMissingValues(boolean newMatchMissingValues)
```

:   Sets whether missing values are counted as a match.

    :   **Parameters:**: `newMatchMissingValues` - true if missing values are counted as a match.

---


### invertSelectionTipText

```
public java.lang.String invertSelectionTipText()
```

:   Returns the tip text for this property

    :   **Returns:**: tip text for this property suitable for displaying in the explorer/experimenter gui

---


### getInvertSelection

```
public boolean getInvertSelection()
```

:   Get whether the supplied columns are to be removed or kept

    :   **Returns:**: true if the supplied columns will be kept

---


### setInvertSelection

```
public void setInvertSelection(boolean invert)
```

:   Set whether selected values should be removed or kept. If true the
    selected values are kept and unselected values are deleted.

    :   **Parameters:**: `invert` - the new invert setting

---


### nominalIndicesTipText

```
public java.lang.String nominalIndicesTipText()
```

:   Returns the tip text for this property

    :   **Returns:**: tip text for this property suitable for displaying in the explorer/experimenter gui

---


### getNominalIndices

```
public java.lang.String getNominalIndices()
```

:   Get the set of nominal value indices that will be used for selection

    :   **Returns:**: rangeList a string representing the list of nominal indices.

---


### setNominalIndices

```
public void setNominalIndices(java.lang.String rangeList)
```

:   Set which nominal labels are to be included in the selection.

    :   **Parameters:**: `rangeList` - a string representing the list of nominal indices. eg: first-3,5,6-last **Throws:**: `InvalidArgumentException` - if an invalid range list is supplied

---


### setDontFilterAfterFirstBatch

```
public void setDontFilterAfterFirstBatch(boolean b)
```

:   Set whether to apply the filter to instances that arrive once
    the first (training) batch has been seen. The default is to
    not apply the filter and just return each instance input. This
    is so that, when used in the FilteredClassifier, a test instance
    does not get "consumed" by the filter and a prediction is always
    generated.

    :   **Parameters:**: `b` - true if the filter should \*not\* be applied to instances that arrive after the first (training) batch has been processed.

---


### getDontFilterAfterFirstBatch

```
public boolean getDontFilterAfterFirstBatch()
```

:   Get whether to apply the filter to instances that arrive once
    the first (training) batch has been seen. The default is to
    not apply the filter and just return each instance input. This
    is so that, when used in the FilteredClassifier, a test instance
    does not get "consumed" by the filter and a prediction is always
    generated.

    :   **Returns:**: true if the filter should \*not\* be applied to instances that arrive after the first (training) batch has been processed.

---


### dontFilterAfterFirstBatchTipText

```
public java.lang.String dontFilterAfterFirstBatchTipText()
```

:   Returns the tip text for this property.

    :   **Returns:**: tip text for this property suitable for displaying in the explorer/experimenter gui

---


### setNominalIndicesArr

```
public void setNominalIndicesArr(int[] values)
```

:   Set which values of a nominal attribute are to be used for
    selection.

    :   **Parameters:**: `values` - an array containing indexes of values to be used for selection **Throws:**: `InvalidArgumentException` - if an invalid set of ranges is supplied

---


### getRevision

```
public java.lang.String getRevision()
```

:   Returns the revision string.

    :   **Specified by:**: `getRevision` in interface `RevisionHandler` **Overrides:**: `getRevision` in class `Filter`
    :   **Returns:**: the revision

---


### main

```
public static void main(java.lang.String[] argv)
```

:   Main method for testing this class.

    :   **Parameters:**: `argv` - should contain arguments to the filter: use -h for help


---


|  |  |  |
| --- | --- | --- |
| **Overview** \| **Package** \| **Class** \| **Tree** \| **Deprecated** \| **Index** \| **Help** \| **Weka's home** | |  |
| **PREV CLASS**   **NEXT CLASS** | **FRAMES**    **NO FRAMES**     **All Classes** |  |
| SUMMARY: NESTED \| FIELD \| CONSTR \| METHOD | DETAIL: FIELD \| CONSTR \| METHOD |  |


---
